# PHF8 promotes osteogenic differentiation of BMSCs in old rat with osteoporosis by regulating Wnt/β-catenin pathway

**DOI:** 10.1515/biol-2022-0523

**Published:** 2022-12-02

**Authors:** Feng Pan, Kai Huang, Hongbin Dai, Chunhe Sha

**Affiliations:** Department of Orthopaedics Part 2, Shanghai Jing’an District Zhabei Central Hospital, No. 619, Zhonghua New Road, Jing’an District, Shanghai, 200073, China

**Keywords:** osteoporosis, PHF8, osteogenic differentiation, old age, Wnt/β-catenin signaling pathway

## Abstract

Osteoporosis is a progressive bone disorder with a higher incidence in the elderly and has become a major public health concern all over the world. Therefore, it is urgent to investigate the mechanisms underlying the pathogenesis of osteoporosis. In this study, the osteoporosis animal model was established, and then rat bone marrow mesenchymal stem cells (rBMSCs) were cultured. The results showed that PHF8 expression was decreased in osteoporosis rats compared to controls. Overexpression of PHF8 promoted BMSC osteogenic differentiation and the expression of osteogenesis-related genes. In addition, the Wnt/β-catenin signaling pathway in BMSCs was inhibited in osteoporosis rats, which was rescued by overexpression of PHF8. After treatment with the Wnt pathway antagonist, the improved osteogenic differentiation of BMSCs induced by overexpression of PHF8 was blocked. Collectively, our data revealed that the decreased expression of PHF8 in osteoporosis rats suppressed the osteogenic differentiation of BMSCs, which was then restored by PHF8 overexpression. Furthermore, the inhibition of the Wnt/β-catenin signaling pathway in BMSCs suppressed osteogenic differentiation. Thus, these findings indicated that PHF8 plays a role in osteogenic differentiation through the Wnt/β-catenin signaling pathway.

## Graphical abstract

**Figure:**
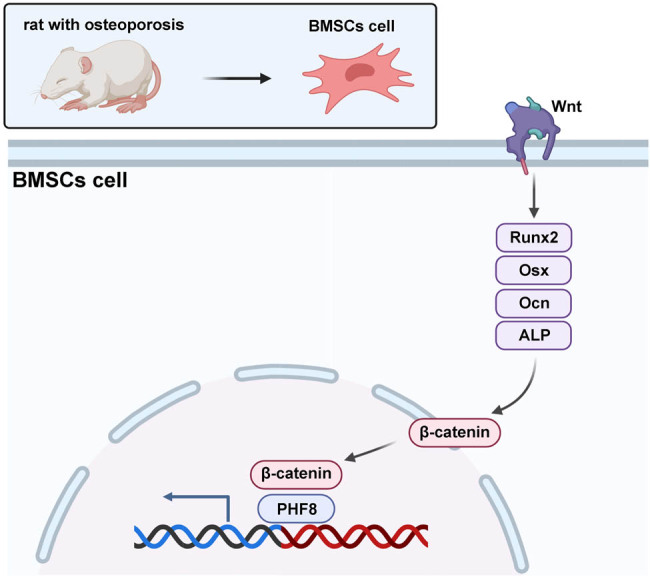

## 1 Introduction

Osteoporosis is a major clinical problem in the elderly population worldwide [1]. The epidemiological survey indicates that more than 200 million people suffer from osteoporosis, and its incidence increases with age [2,3]. Osteoporosis affects more than 70% of people over the age of 80 and is more common in women than men. In developed countries, the morbidity of osteoporosis is about 2–8% for men and 9–38% for women [4]. There are approximately 9 million osteoporosis-related fractures every year worldwide. Therefore, it is significantly important to elucidate the mechanisms underlying the pathogenesis of osteoporosis, which may provide new therapeutic targets.

Bone marrow mesenchymal stem cells (BMSCs) are considered to be multipotential progenitor cells with strong proliferation ability that are able to differentiate into various types of cells, including osteoblasts, chondrocytes, adipocytes, osteocytes, and myoblasts [5]. BMSCs have been recently found to play a crucial role in bone development, remodeling, and regeneration [6]. The capacity of BMSCs to undergo osteogenic differentiation is decreased during osteoporosis, resulting in a defect in bone formation [7]. It has been reported that the disturbed homeostasis of BMSC adipogenesis and osteogenesis contributes to osteoporosis [8]. However, the mechanism for abnormal BMSC osteogenic differentiation remains elusive.

Activation of Wnt/β-catenin signaling pathways can regulate cell proliferation and survival [9]. When the Wnt/β-catenin signaling pathway is activated, the amount of β-catenin increases and it accumulates in the nucleus, where it interacts with specific transcription factors to regulate the transcription of target genes. Inappropriate activation of the Wnt/β-catenin signaling pathway is associated with various types of tumors [10,11,12,13], obesity [14], and neurodegenerative diseases [15], as well as bone diseases such as osteoarthritis and osteoporosis [16, 17]. Computational modeling is wildly used to investigate complex signaling networks and predict the function of compounds [18,19,20]. *In-silico* studies provide insight into the effect of each compound and reveal which perturbations may deregulate the basal healthy state of cells and tissues [21,22]. Structurally, Dickkopf-1 (DKK-1) has been proven to serve as the antagonist for the Wnt signaling pathway [23], which participates in the regulation of bone metastases and carcinogenesis [24,25].

In this study, we aimed to explore the role of PHF8 in osteoporosis. Through ovariectomy, we constructed an animal model of osteoporosis and isolated BMSCs from rat femur and tibial epiphyseal regions for *in vitro* cell experiments. Using an animal model of osteoporosis and *in vitro* BMSCs culture, we examined the expression of PHF8 and the osteogenesis efficiency of BMSC. We found that PHF8 regulated osteogenic differentiation in rat bone marrow mesenchymal stem cells (rBMSCs) via the Wnt/β-catenin signaling pathway. Then this study identified a new target, PHF8, regulating BMSC osteogenic differentiation, providing crucial insight into the mechanism underlying the pathogenesis of osteoporosis and new therapeutic options for osteoporosis.

## 2 Materials and methods

### 2.1 Animal model establishment

Female Wistar rats were purchased from Beijing Huafukang Bioscience Co., Inc (Beijing, China) and housed at specific pathogen-free (SPF) conditions.

Twelve female SPF Wistar rats (aged 7 months and weighing 400–500 g) were randomly divided into sham and ovariectomy (OVX) groups. A bilateral ovariectomy was performed to establish an osteoporosis model for the rats in the OVX group, while a sham surgery in which the ovaries of rats were only exteriorized but not resected was carried out on the sham group as previously described [26,27]. Six weeks after surgery, the femurs of rats from the sham and OVX groups were collected.


**Ethical approval:** The research related to animal use has been complied with all the relevant national regulations and institutional policies for the care and use of animals and was approved by the Ethics Committee of the Shanghai Jing’an District Zhabei Central Hospital.

### 2.2 Cell culture and transfection

Rat BMSCs (rBMSCs) were obtained from the femur and tibial epiphyseal regions of rats as previously described [28]. rBMSCs were transfected with a PHF8-overexpression plasmid vector (GeneChem, Shanghai, China) and control vectors as previously described [29]. When rBMSCs reached 60–80% confluence, cells were transfected with Lipofectamine transfection reagent (Thermo Fisher Scientific, Waltham, MA, USA) and plasmid DNA for 12 h according to the manufacturer’s protocol.

### 2.3 Histological and Alizarin Red S staining

The femurs of rats from the sham and OVX groups were fixed with 4% paraformaldehyde, decalcified with 0.5 M ethylenediamine tetraacetic acid, embedded in paraffin blocks, and sectioned at a thickness of 4–5 μm. Then, hematoxylin and eosin (H&E) staining was performed on the sections [30].

For cellular immunofluorescence (IF), the antibody against β-catenin (ab32572; Abcam, Cambridge, MA, UK) was used. Cells were stained with 4’,6-diamidino-2-phenylindole reagent for nuclear staining.

Osteogenesis was detected by Alizarin Red S (Sigma–Aldrich, St Louis, MO, USA) as previously described [31]. Briefly, cells were washed with phosphate-buffered saline and soaked in 2% Alizarin Red for 30 min at room temperature, then imaged as described earlier.

Staining was observed by microscopic (Olympus, Japan) and photographed. Histological results were analyzed by ImageJ software.

### 2.4 Western blotting

The cells and tissue homogenate were lysed in radio-immunoprecipitation assay buffer with protease and phosphatase inhibitors. The concentrations of the supernatant were determined by a bicinchoninic acid (BCA) protein detection kit (Servicebio, Wuhan, China) [32]. Equal amounts of protein were loaded, separated by 10% SDS–PAGE, and transferred to polyvinylidene fluoride membranes. After blocking, the membrane was incubated with anti-PHF8, Runt-related transcription factor-2 (RUNX2), alkaline phosphatase (ALP), osterix (Osx), osteopontin (OPN), lamin B1, Wnt, β-catenin, and glyceraldehyde-3-phosphate dehydrogenase (GAPDH) antibodies. The detailed information on antibodies used in western blotting is listed in Table 1.

**Table 1:** Primary antibodies used in Western blotting

Antibody	Species	Concentration	Reference	Source
PHF8	Rabbit	1:1,000	ab280887	Abcam (Cambridge, MA, UK)
RUNX2	Rabbit	1:1,000	ab236639	Abcam
ALP	Rabbit	1:1,000	ab133602	Abcam
Osx	Rabbit	1:1,000	ab209484	Abcam
OPN	Rabbit	1:1,500	ab63856	Abcam
Wnt5a	Rabbit	1:1,000	ab227229	Abcam
β-Catenin	Rabbit	1:1,000	ab32572	Abcam
Lamin B1	Rabbit	1:2,000	PB9611	Boster Biological Technology (Wuhan, China)
GAPDH	Mouse	1:2,000	BM1623	Boster Biological Technology

### 2.5 Real-time polymerase chain reaction (PCR)

Total RNA was extracted from cultured cells using Trizol reagent (TaKaRa Biotechnology, Dalian, China) according to the manufacturer’s instructions [33]. The cDNA was generated using the PrimerScriptTM RT reagent Kit (TaKaRa Biotechnology). Real-time PCR was performed with StepOnePlus (Applied Biosystems, Foster City, CA, USA) and using SYBR Premix Ex Taq kit (TaKaRa Biotechnology). Relative gene expression was calculated using the 2(-Delta CT) method and normalized to GAPDH. Primer sequences are listed in Table 2.

**Table 2:** Primers used in real-time PCR

Primers	Primer sequences
PHF8	(F) 5′-TCCTCTCTGTGCTGGTGTTT-3′
	(R) 5′-AGGTCAGGAAGCATTGTGGA-3′
RUNX2	(F) 5′-ACCTCCAGGAAGCCTTTGAT-3′
	(R) 5′-CCTGGTGGTGTCACTGAATG-3′
ALP	(F) 5′-CCTTCTTCCGTCAGTACCGT-3′
	(R) 5′-AGCTGGTTCATCCCGATTGT-3′
Osx	(F) 5′-ATTCTCCCTCCCTCTCCCTT-3′
	(R) 5′-TGGAAGTGAGTAGCAGTGCA-3′
OPN	(F) 5′-GAGGAGAAGGCGCATTACAG-3′
	(R) 5′-ACAGAATCCTCGCTCTCTGC-3′
GAPDH	(F) 5′-TGCTGAGTATGTCGTGGAG-3′
	(R) 5′-GCATCAAAGGTGGAAGAAT-3′

### 2.6 ALP activity

ALP activity was detected in the cell supernatants using a colorimetric assay kit (Jiancheng, Nanjing, China) [34]. ALP activity was examined at 405 nm and calculated with a standard curve.

### 2.7 Statistical analysis

All data analysis and visualization were carried out using GraphPad Prism software (version 9.0). The data were expressed as the mean ± standard deviation of at least three experimental replicates. A Shapiro–Wilkinson test was used to confirm the normality of the data. If the data were normally distributed, ANOVA with Tukey’s *post hoc* test for multiple comparisons and a Student’s *t*-test for binary comparisons was applied. Otherwise, a Kruskal–Wallis test with Dunn’s *post hoc* test for multiple comparisons and a Mann–Whitney *U*-test for binary comparisons were performed. *P* < 0.05 was considered statistically significant.

## 3 Results

### 3.1 The downregulated expression of PHF8 in the OVX group

**Figure 1:**
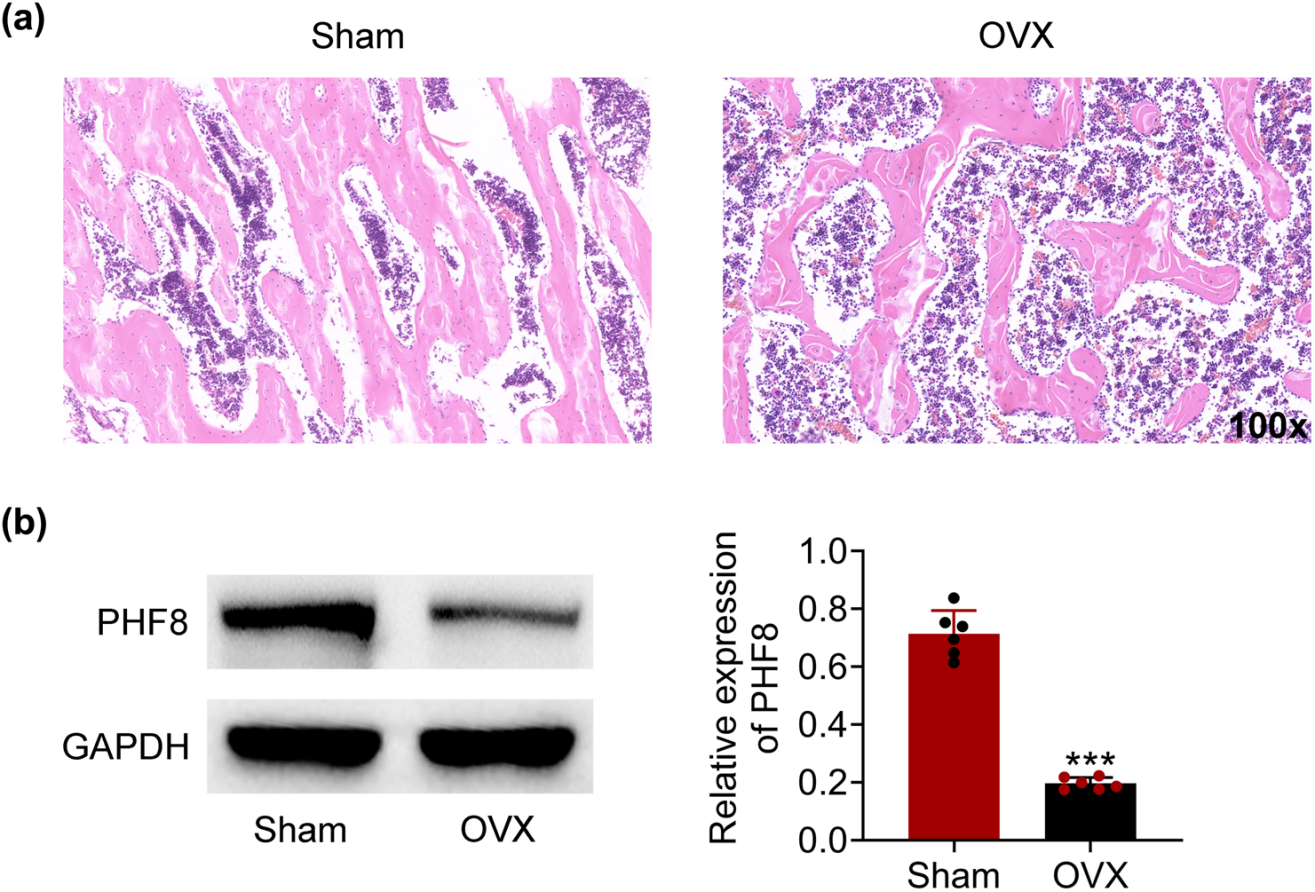
The down-regulated expression of PHF8 in the OVX group. (a) H&E staining of bone microarchitectures in the femur showing a bone mass loss in the OVX group versus sham groups. (b) The protein level of PHF8 in the OVX and sham groups was detected by Western blotting. ***P < 0.001 versus the sham group.

To confirm the establishment of the osteoporosis rat model, bone microarchitectures in the femur were examined by H&E staining. We found that trabecular bone mass was markedly decreased in the OVX group compared with sham groups (Figure 1a). Furthermore, the protein level of PHF8 was downregulated in the OVX group in comparison to the sham group, which suggested that PHF8 played a role in the osteoporosis of rat (Figure 1b).

### 3.2 PHF8 promotes the osteogenesis efficiency of BMSCs

**Figure 2:**
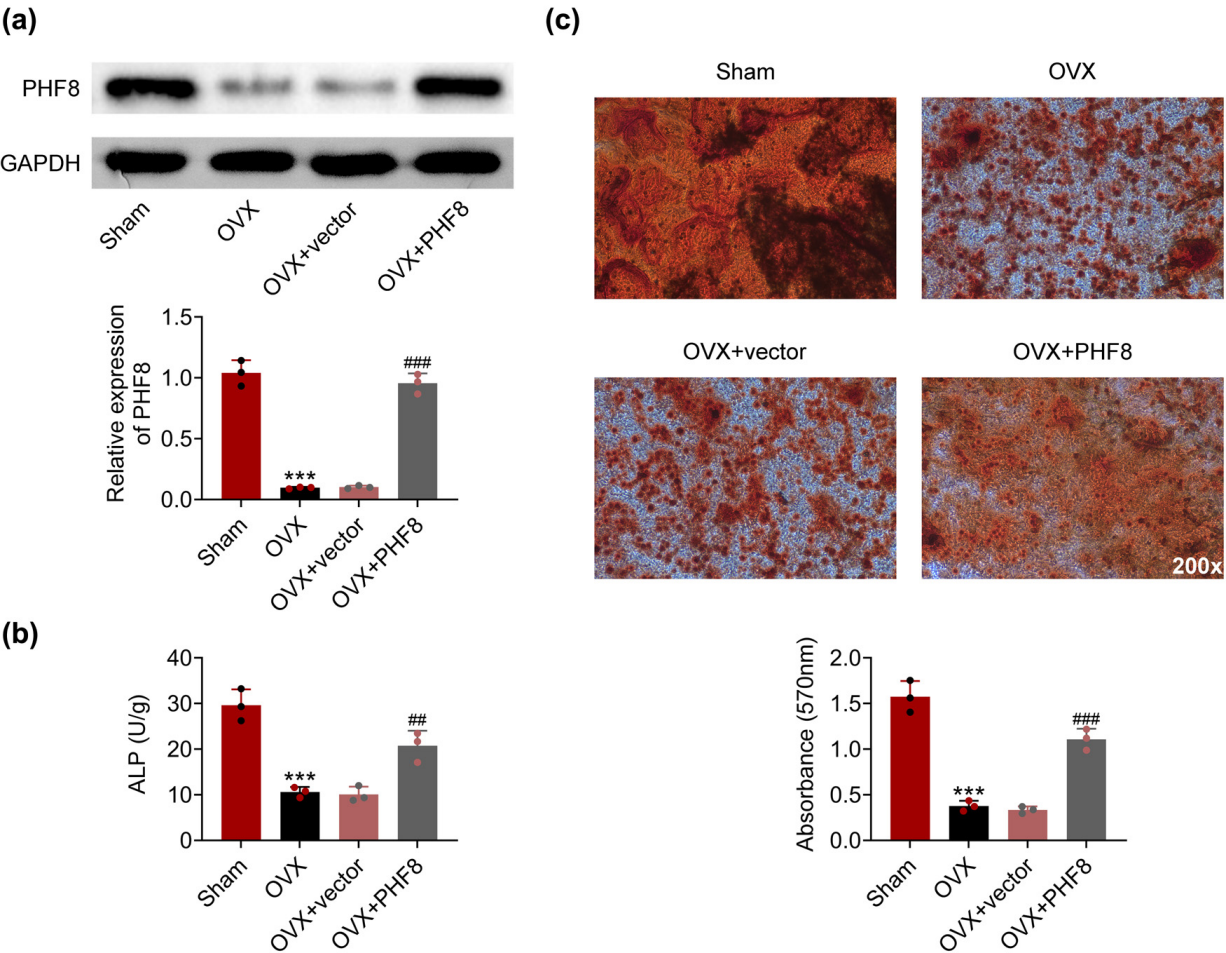
PHF8 promotes the osteogenesis efficiency of BMSCs. (a) The protein level of PHF8 in rBMSCs was detected by western blotting. (b) The activity of ALP was detected by ELISA. (c) The osteogenesis efficiency of BMSCs was measured by the Alizarin Red S staining. ***P < 0.001 versus the sham group; ^##^ *P* < 0.01 and ^###^ *P* < 0.001 versus the OVX group.

To evaluate the function of PHF8, rBMSCs were isolated and transfected with a PHF8 overexpression plasmid vector. After transfection, the increased expression of PHF8 in rBMSCs was confirmed by Western blotting (Figure 2a). Of significance, the activity of ALP was reduced in the OVX group compared with the sham group, while overexpression of PHF8 in rBMSCs reversed this decrease (Figure 2b). In addition, Alizarin Red S staining results showed that overexpression of PHF8 improved rBMSC osteogenic differentiation, which was inhibited in the OVX group (Figure 2c). Altogether, these data implied that overexpression of PHF8 in rBMSC reversed the inhibition of rBMSC osteogenic differentiation in the OVX group.

### 3.3 PHF8 enhances the expression of key factors associated with the osteogenic differentiation of BMSCs

**Figure 3:**
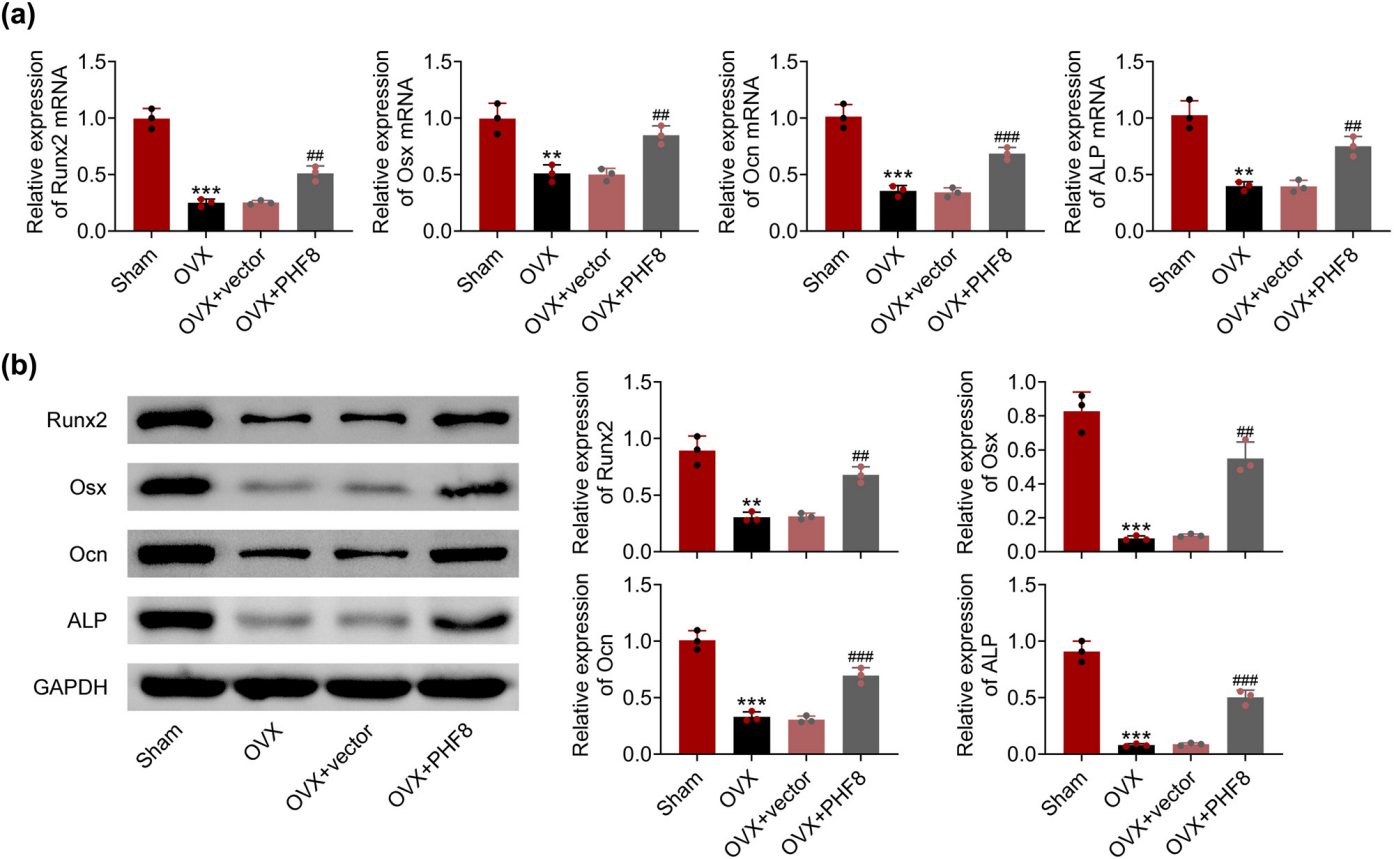
PHF8 enhances the expression of key factors related to osteogenic differentiation of BMSCs. (a) The relative mRNA levels of RUNX2, Osx, Ocn, and ALP in BMSCs detected by real-time PCR. (b) The relative protein levels of RUNX2, Osx, Ocn, and ALP in BMSCs detected by Western blotting. ***P < 0.001 versus the sham group; ^##^ *P* < 0.01 and ^###^ *P* < 0.001 versus the OVX group.

To further investigate the effect of PHF8 on rBMSCs, the mRNA and protein expression levels of key factors related to osteogenic differentiation were detected by real-time PCR (Figure 3a) and western blotting (Figure 3b), respectively. In the OVX group, the expressions of RUNX2, Osx, Ocn, and ALP in BMSCs were downregulated compared to the sham group. Overexpression of PHF8 upregulated the expression of RUNX2, Osx, Ocn, and ALP in BMSCs, revealing the role of PHF8 in osteogenic differentiation.

### 3.4 PHF8 activates Wnt/β-catenin signaling pathways

**Figure 4:**
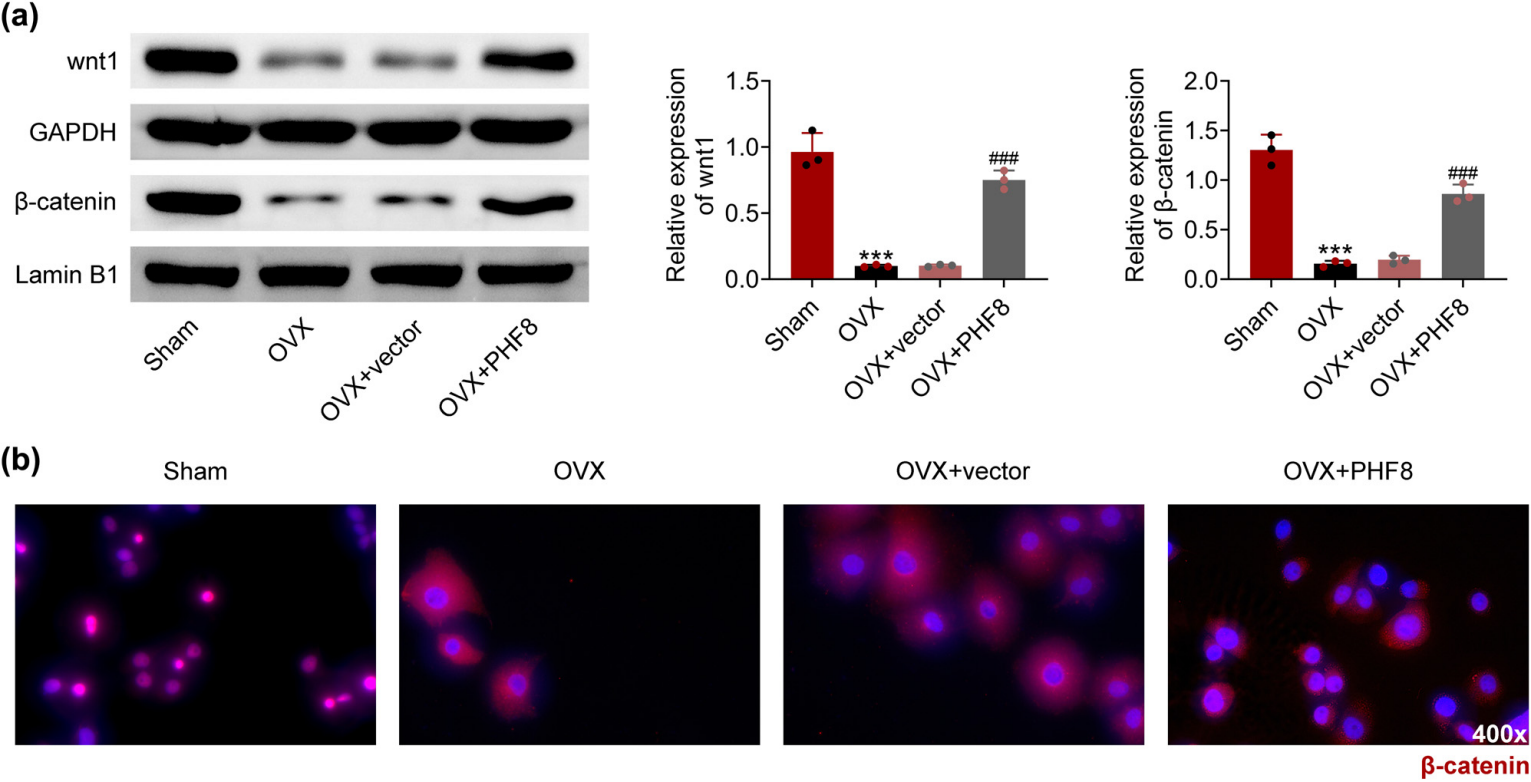
PHF8 activates Wnt/β-catenin signaling pathway. (a) The relative protein levels of Wnt and β-catenin in BMSCs detected by western blotting. (b) The β-catenin positive cells in BMSCs showed by immunofluorescence. ***P < 0.001 versus the sham group; ^###^ *P* < 0.001 versus the OVX group.

The Wnt/β-catenin signaling pathway plays a fundamental role in bone remodeling, such as in osteoporosis [17]. Whether the Wnt/β-catenin signaling pathway is affected by PHF8 needs to be explored. In the OVX group, Wnt and β-catenin expressions in rBMSCs were decreased compared to the sham group, which was increased by overexpression of PHF8 (Figure 4a). IF showed that β-catenin positive BMSCs were elevated in the OVX group with PHF8 overexpression compared to the OVX group (Figure 4b).

### 3.5 PHF8 induces osteogenic differentiation of BMSCs via the Wnt/β-catenin signaling pathway

**Figure 5:**
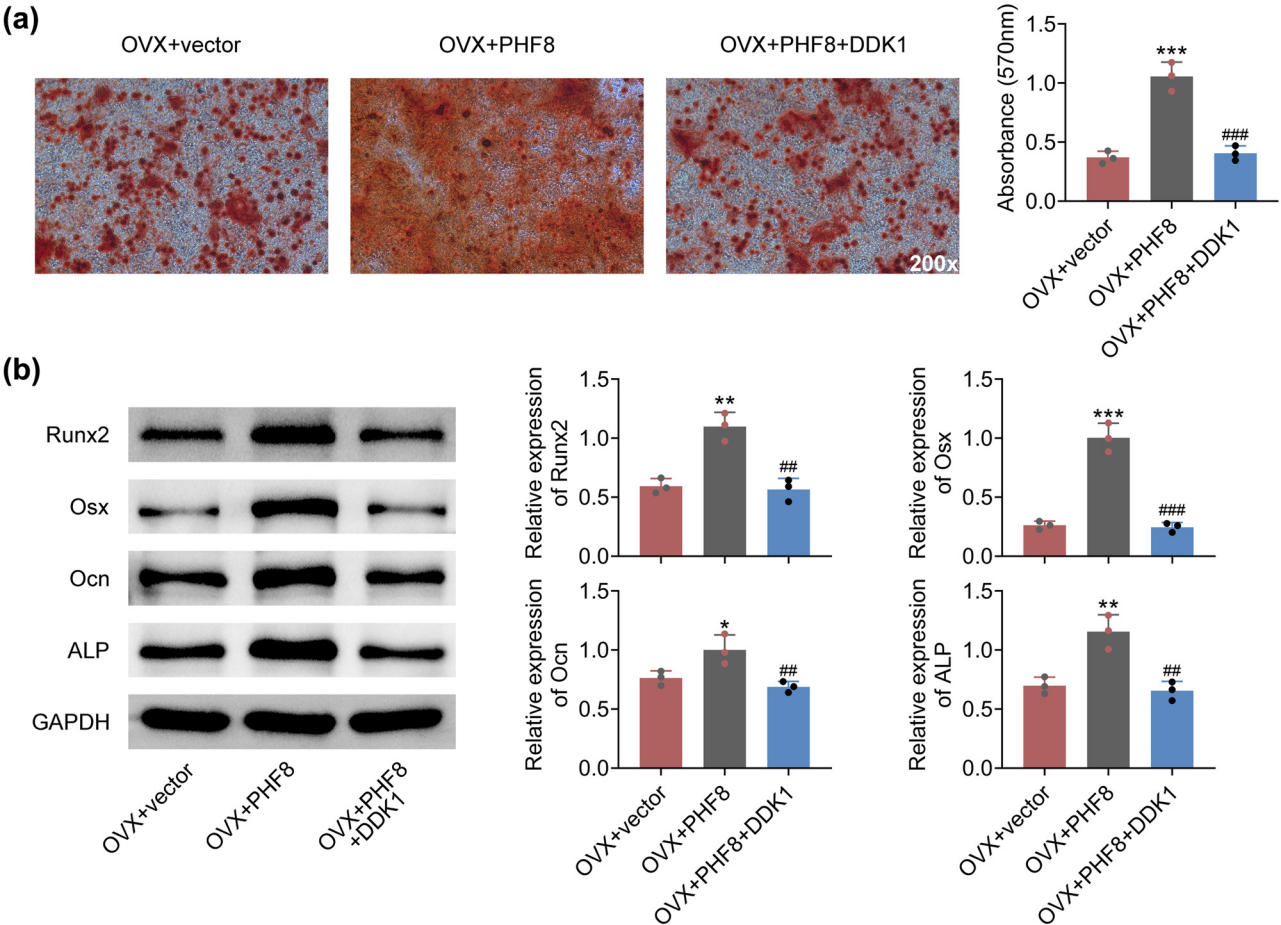
PHF8 induces osteogenic differentiation of BMSCs via Wnt/β-catenin signaling pathway. (a) The osteogenesis efficiency of BMSCs was measured by the Alizarin Red S staining. (b) The relative protein levels of RUNX2, Osx, Ocn, and ALP in BMSCs detected by Western blotting. *P < 0.05, **P < 0.01 and ***P < 0.001 versus the sham group; ^###^ *P* < 0.001 versus the OVX group.

To further explore the mechanisms of PHF8 underlying osteogenic differentiation, BMSCs were treated with the Wnt pathway antagonist, DDK1. Alizarin Red S staining results indicated that treatment with DDK1 weakened rBMSC osteogenic differentiation induced by overexpression of PHF8 (Figure 5a). Meanwhile, the upregulated expressions of RUNX2, Osx, Ocn, and ALP induced by overexpression of PHF8 in BMSCs were downregulated by DDK1 treatment (Figure 5b). Taken together, these data suggested that PHF8 regulated the osteogenic differentiation of BMSCs via the Wnt/β-catenin signaling pathway.

## 4 Discussion

Osteoporosis is a progressive bone disorder with an increasing incidence worldwide, especially in the elderly, and it is characterized by the systemic loss of bone mass and microarchitecture, which increases the risk of fragility fractures [1,2,3,35]. These fractures are linked to increased healthcare costs, physical disability, poor quality of life, and mortality. In this study, we demonstrated the downregulated expression of PHF8 in the OVX group. Overexpression of PHF8 promoted the osteogenic differentiation of BMSCs. In addition, overexpression of PHF8 activated the Wnt/β-catenin signaling pathway in BMSCs. Moreover, treatment with the Wnt pathway antagonist inhibited the effect of PHF8 on BMSCs. Therefore, PHF8 plays a role in the osteogenic differentiation of BMSCs via the Wnt/β-catenin signaling pathway and could be a potential therapeutic target for osteoporosis.

Histone demethylases have emerged as key regulators of biological processes, and the disorder of histone demethylases is associated with a variety of cancers [36], neuronal diseases [37], and cardiac diseases [38]. PHF8, one of the histone demethylases, is upregulated in some types of tumors, such as neuroendocrine prostate cancer [39], non-small-cancer lung carcinoma [40], hepatocellular carcinoma [41], gastric cancer [42], and breast cancer [43]. PHF8 plays a crucial role in a variety of biological processes [44]. In this study, we found a reduction of PHF8 expression in osteoporosis rats. Osteoporosis is featured by a progressive loss of bone mass and an accumulation of fat in the bone marrow [1]. The disturbed homeostasis of BMSC adipogenesis and osteogenesis could contribute to osteoporosis [27]. We found that overexpression of PHF8 in rBMSC promoted osteogenic differentiation, suggesting that PHF8 might play a role in osteoporosis by regulating rBMSC osteogenic differentiation.

The Wnt/β-catenin signaling pathway has been found to regulate a series of biological processes [9]. Inappropriate activation of the Wnt/β-catenin pathway is associated with various types of cancers [11] and neurodegenerative diseases [45], as well as bone diseases such as osteoarthritis [16] and osteoporosis [31]. It has been reported that BMSC-derived exosomes alleviate the osteogenesis of osteoporosis via the Wnt/β-catenin signaling pathway [46]. In this study, the Wnt/β-catenin signaling pathway was inhibited in rBMSC derived from osteoporosis rats compared to control rats. According to previous studies, PHF8 occupied the Wnt1 promoter, leading to a decrease in repressive histone in the promoter region of the Wnt1 gene, which in turn promoted its transcription [40]. Consistent with this result, our results showed that after transfection with the PHF8 overexpression plasmid vector, the inhibition of Wnt/β-catenin signaling was reversed in rBMSC derived from osteoporosis rats. Moreover, after treatment with the Wnt pathway antagonist, the effect of PHF8 on osteogenic differentiation was blocked, which suggested that the ability of PHF8 is triggered by the Wnt/β-catenin signaling pathway. Here, we identified that PHF8 played a protective role in osteoporosis by affecting osteogenic differentiation via the Wnt/β-catenin signaling pathway.

There are several limitations to this study. The major limitations of this study were the lack of human specimens that could be used to further verify the effect of PHF8 on human BMSCs. Although animal and cell experiments may closely mimic the characteristics of osteoporosis disease, the consistency would be enhanced if further verification is conducted. While this report was the result of preliminary research, we will utilize human specimens in further research.

In summary, we first found that decreased PHF8 expression deturbed osteogenic differentiation of BMSCs and inhibited the Wnt/β-catenin signaling pathway in osteoporosis rats. In addition, PHF8 overexpression promoted osteogenic differentiation of BMSCs, which was suppressed by inhibition of the Wnt/β-catenin signaling pathway. Thus, these results uncovered that PHF8 perform its promoting function of osteogenic differentiation via the Wnt/β-catenin signaling pathway. Thereby, this study provides novel insights into the mechanisms underlying the development of osteoporosis, which may provide a potential therapeutic target in the future.
